# Extensive Condyloma Lata Lesions in Unusual Sites: An Atypical Manifestation of Secondary Syphilis

**DOI:** 10.1155/carm/8595258

**Published:** 2025-09-25

**Authors:** Retno Hesty Maharani, Nur Mala Il Ala, Rasmia Rowawi, Eva Krishna Sutedja, Inne Arline Diana, Laila Tsaqilah, Hermin Aminah Usman, Pati Aji Achdiat

**Affiliations:** ^1^Department of Dermatology and Venereology, Faculty of Medicine, Universitas Padjadjaran-Dr. Hasan Sadikin General Hospital, Bandung, Indonesia; ^2^Department of Anatomical Pathology, Faculty of Medicine, Universitas Padjadjaran-Dr. Hasan Sadikin General Hospital, Bandung, Indonesia

**Keywords:** extragenital lesions, great imitator, human immunodeficiency virus infection, penicillin therapy, sexually transmitted infection

## Abstract

Condyloma lata is a typical lesion of secondary syphilis, which usually occurs in the flexural and anogenital areas. Secondary syphilis can be misdiagnosed with atypical clinical manifestations that can mimic other skin diseases (the great imitator), especially in patients with human immunodeficiency virus (HIV) infection. Atypical condyloma lata lesions can occur outside the flexural and extragenital areas. Therefore, it can mimic other skin diseases. This case report aims to report a case of secondary syphilis in the form of extensive condyloma lata lesions in unusual sites in a 46-year-old man with HIV Stage II. Physical examination revealed flat papules and plaques on the face, trunk, and anogenital area; papules with excoriations on the extremities; and mucous patches. *Treponema pallidum* hemagglutination assay and Venereal Disease Research Laboratory (VDRL) titer were 1:5120 and 1:256, respectively. The *Chlamydia trachomatis* and hepatitis C examination, as well as CD4+ T cell count and HIV viral loads data, were unavailable for this patient. Histopathological findings from flat papules on the patient's face, abdomen, and inguinal region showed eroded epidermis with neutrophil infiltration, spongiotic reactions, acanthosis, parakeratosis, hypogranulosis, superficial pallor, and endarteritis with the infiltration of plasmocytes and polymorphonuclear cells, which support that the patient's lesion is condyloma lata. The patient was treated with a single dose of 2.4 million units of benzylpenicillin G intramuscularly. The result of VDRL titer examination after 3 months of therapy was 1:32, with improvement in the skin lesions seen as the lesions that became hyperpigmentation macules. This case report showed condyloma lata, a hallmark of secondary syphilis, can present as extensive lesions in extragenital sites. Awareness of the atypical manifestations is essential to ensure early diagnosis through histopathological and serological evaluation.

## 1. Introduction

Syphilis is a disease caused by *Treponema pallidum* (*T. pallidum*) subspecies pallidum. This infection is mainly transmitted through sexual contact [1]. Syphilis is still a problem because its incidence continues to increase every year, especially in men who have sex with men (MSM) and patients with human immunodeficiency virus (HIV) infection [2]. Syphilis is easy to treat, but in a latent stage without symptoms or secondary syphilis with atypical clinical manifestations that resemble other skin diseases (the great imitator), this disease often goes undiagnosed [3].

Secondary syphilis typically presents with a nonpruritic macular or papular exanthem that predominantly affects the trunk and extremities, with frequent involvement of the palms and soles. Additional well-recognized but less common features include condyloma lata, which appear as well-defined hypertrophic papules or plaques with a flat surface [4, 5]. Atypical manifestations of secondary syphilis are all manifestations that differ from the typical ones described above in morphology, number, localization, and associated symptoms [4]. Condyloma lata has a predilection in the flexural areas, especially in the anogenital areas. Atypical extragenital condyloma lata have been reported at multiple sites including the face [6–8], scalp [9], nape [10], trunk [11], ankle [12], and oral mucosa [13].

Examinations that are often used to establish the diagnosis of syphilis are treponemal serological tests and nontreponemal serological tests. Histopathological examination can be performed to help establish the diagnosis of syphilis that is not typical or doubtful [1]. This case report aims to present a case of atypical secondary syphilis as extensive condyloma lata lesions that occurred in unusual sites, which are not only in the genital area but also in the extragenital area, including the face, chest, and abdomen, in an MSM patient with HIV infection.

## 2. Case Presentation

A 46-year-old man presented with chief complaints of itchy raised red lesions that appeared on the face, chest, and abdomen. The lesions first appeared 4 months before the consultation in the form of erythematous macules on the face, chest, and abdomen, accompanied by itching. Two weeks later, the erythematous macules became erythematous papules and plaques. The patient had no known history of primary syphilis or any symptoms of neurosyphilis and ocular syphilis. History of prior similar signs and symptoms or any sexually transmitted infections (STIs) was denied. The patient was married at the age of 24 and had sexual intercourse for the first time after marriage. The patient had sex with his male friend 6 years ago and another male friend without a condom 6 months before the appearance of the first skin lesion. The patient also admitted to a weight loss of 4 kg (6%) over the past 3 months. There were no other systemic symptoms. The patient denied having tattoos and consuming any alcohol or drugs.

**Figure 1:**
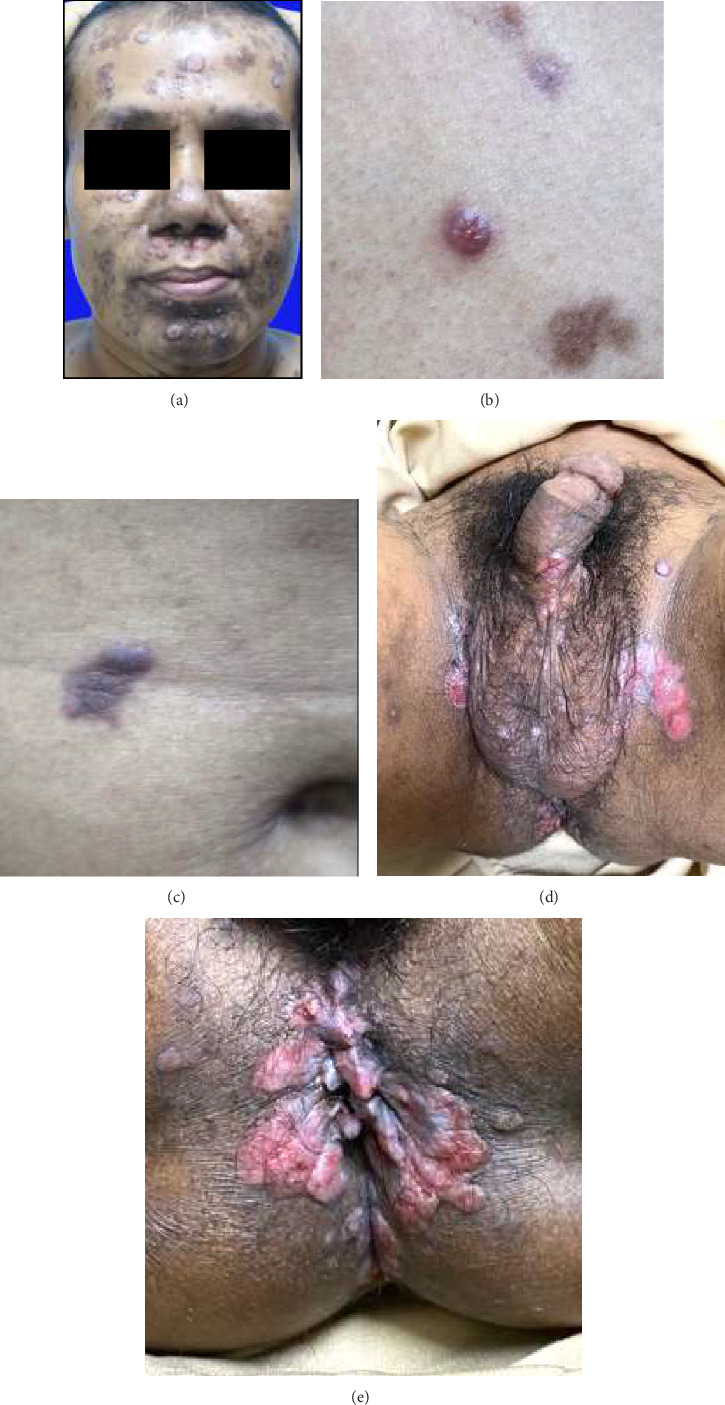
Clinical image of skin lesions showed flat papule and plaques on the face (a), chest (b), abdomen (c), genitalia (d), and perianal areas (e).

**Figure 2:**
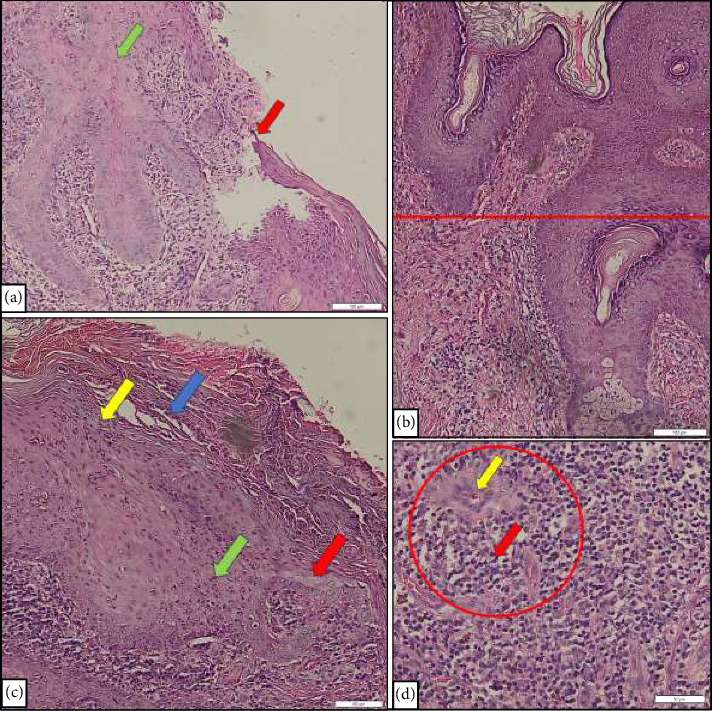
Histopathological examination from flat papule lesions suspected of being condyloma lata lesions on the face, abdomen, and inguinal showed eroded epidermis (red arrow) and spongiosis (green arrow) (a); irregular acanthosis (red line) (b); parakeratosis (blue arrow), hypogranulosis (yellow arrow), neutrophil infiltration (green arrow), and superficial pallor (red arrow) (c); as well as endarteritis (yellow arrow) and dense inflammatory infiltrates consisting of plasmocytes and polymorphonuclear cells (red circle and red arrow) (d).

Clinical examination revealed mucous patches on the hard palate. The other general examination was within normal limits. There were well-defined erythematous and hyperpigmented flat papules and plaques on the face, chest, and abdomen (Figures 1(a), 1(b), and 1(c)). On the pubis, the body of the penis, glans penis, scrotum, perineum, anus, and perianal, there were moist erythematous plaques with flat surfaces and scales (Figures 1(d) and 1(e)). No Gram-negative diplococci or *Trichomonas vaginalis* were observed on direct microscopic examination of the urethral swab. The laboratory examination revealed a reactive anti-HIV test with *T. pallidum* hemagglutination assay (TPHA) titer 1:5120 and Venereal Disease Research Laboratory (VDRL) titer 1:256 and nonreactive hepatitis B surface antigen (HBsAg). The *Chlamydia trachomatis* and hepatitis C examination, as well as CD4+ T cell count and HIV viral loads data, were unavailable for this patient, as these tests had not been performed due to the limitations of the facility in the COVID-19 era. The histopathological examinations from flat papule lesions suspected of being condyloma lata lesions on the patient's face, abdomen, and inguinal revealed eroded epidermis with neutrophil infiltration, spongiotic reactions, acanthosis, parakeratosis, hypogranulosis, superficial pallor, and endarteritis with the infiltration of plasmocytes and polymorphonuclear cells, which support that the patient's lesion is condyloma lata (Figure 2). The periodic acid–Schiff staining was performed to rule out the deep mycoses infection, as the differential diagnosis and the result were negative. Thus, the patient was diagnosed with HIV stage II with secondary syphilis.

**Figure 3:**
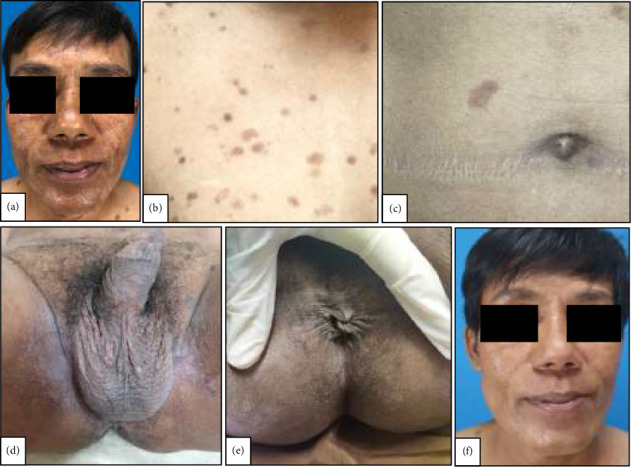
Clinical image of the skin lesions 3 months posttherapy showed hyperpigmented macules on the face (a), chest (b), abdomen (c), genitalia (d), and perianal areas (e). At 5 months posttherapy, the hyperpigmented macules on his face had significantly faded (f). Abbreviations: ARV: antiretroviral; BPG: benzylpenicillin G; CDC: Centers for Disease Control and Prevention; FDC: fixed-dose combination; HBsAg: hepatitis B surface antigen; HIV: human immunodeficiency virus; MSM: men who have sex with men; STIs: sexually transmitted infections; TPHA: *Treponema pallidum* hemagglutination assay; VDRL: Venereal Disease Research Laboratory.

A single dose of 2.4 million units of benzylpenicillin G (BPG) intramuscular injection was given to the patient as well as an antiretroviral (ARV) fixed-dose combination (FDC), consisting of tenofovir, lamivudine, and efavirenz once a day, co-trimoxazole 960 mg once a day, and adherence counseling. At 3 months posttherapy, the lesions became hyperpigmented macules, and VDRL titer decreased 8-fold to 1:32 (Figures 3(a), 3(b), 3(c), 3(d), and 3(e)). At 5 months posttherapy, the hyperpigmented macules on his face had significantly faded (Figure 3(f)).

## 3. Discussion

A report from the Centers for Disease Control and Prevention (CDC) stated that there was an increase in new syphilis cases and almost half of them were MSM [2]. Several factors that play a role in increasing the incidence of STIs in MSM are having multiple sexual partners, having sex without a condom, and having anal sex [14]. The risk of STIs will increase with having anogenital sexual intercourse with 3–5 partners in the last 6 months, acting as the receptive, having sex together in a group, and serosorting [15]. The risk of contracting HIV also increases in receptive anogenital sex without a condom, which is 8.2/1000, while the insertive anogenital sex is 0.6/1000 [16]. The patient in this case report is an HIV-diagnosed MSM, who had anogenital sex, acted as the receptive, and did not use condoms.

Syphilis has four clinical stages, namely primary, secondary, latent, and tertiary stages. The hematogenous spread of syphilis in secondary syphilis can cause skin and mucosal lesions [17]. Secondary syphilis is usually characterized by a widespread, nonpruritic exanthem composed of macules and/or papules, typically distributed over the trunk and limbs, with the palms and soles frequently affected [4]. The most common secondary syphilis lesions are diffuse exanthema (54%), macular (16%), maculopapular (16%), papular (12%), papulopustular (2%), and psoriasiform (2%). The most common location of the lesions was on the palms (53%), soles (56%), trunk (26%), genitals (23%), and mucosa (26%) [17]. Only 14%–40% of secondary syphilis lesions are accompanied by itching [18, 19]. Local inflammation as a manifestation of secondary syphilis in the oral mucosa and tongue may form mucous patches [20]. In this case report, the patient had no lesions on the palms and soles, but mucous patches were found on the hard palate. The patient was presented with erythematous macules on the face, chest, and abdomen accompanied by itching. Two weeks later, the erythematous macules became erythematous papules and plaques.

A recent 10-year retrospective study by Ciccarese, et al. [4] reported that 25.8% of the syphilis patients had atypical manifestations, especially in the secondary stage of the disease. Reported variations of atypical secondary syphilis lesions include annular lesions, psoriasiform erythematous scaly plaques, erythematous macules and papules on the hands and feet accompanied by scrotal eczema, balanitis, anetoderma, as well as necrotic and ulcerative lesions [4, 21, 22]. Among these unusual findings, condyloma lata remains a distinctive but less frequent manifestation, occurring in approximately 5%–12% cases of secondary syphilis [18, 19]. Typically arising in flexural regions such as the anogenital area, atypical extragenital presentations of condyloma lata have been reported at various anatomical sites, including the face [6–8], scalp [9], nape [10], trunk [11], ankle [12], and oral mucosa [13].

Pourang, et al. [5] reported condyloma lata lesions as multiple eroded, smooth, pink–white papules on the chest, umbilicus, and popliteal fossa. Sundaraj et al. [8] reported raised, flat papular lesions with widespread distribution on the face that were referred to as condyloma lata lesions based on their appearance in a patient with secondary syphilis. The lesions were accompanied by maculopapular cutaneous lesions all over the body. Extensive condyloma lata lesions at unusual locations had also been reported by Shrivastava et al. [10], which appeared under the chin, nape of the neck, and axilla. The lesion was referred to as condyloma lata, also based on the appearance of the lesions as moist hypertrophic papular lesions in a secondary syphilis patient. Another unusual location of a condyloma lata lesion was on the ankle, which Ikeda et al. [12] reported in 2016. The author referred to the lesions as condyloma lata based on their appearance and the histological findings of irregular acanthosis and papillomatosis, parakeratotic horny layer, and dense lymphocytic and plasma cell infiltration in the papillary and upper dermis. These unusual or widespread lesions of condyloma lata were hypothesized due to mechanical friction and localized excessive sweating. In this case report, the patient presented with condyloma lata on the face, chest, and abdomen, which are unusual sites for this lesion. Therefore, the lesions in the patient were atypical for secondary syphilis. There were also some flat papules and plaques on the genital area. Therefore, further examinations are necessary to obtain the right diagnosis.

In secondary syphilis, a treponemal test, such as TPHA, was performed for screening and confirmation, while the nontreponemal test, such as the VDRL test, was performed for screening and assessing the response to therapy [1]. The patient had a TPHA titer of 1:5120 and a VDRL titer of 1:256; therefore, the patient was diagnosed with secondary syphilis.

The inflammatory cell infiltrates of secondary syphilis may consist of plasmocytes, lymphocytes, and histiocytes. The plasmocyte infiltration was the most common feature (86.4%), followed by acanthosis (72.9%), elongation of the rete ridges (71.2%), swelling of the endothelial cells (69.5%), apoptosis of keratinocytes (69.5%), lichenoid inflammatory pattern (64.4%), and spongiosis (62.5%) [23]. Furthermore, other histological findings of condyloma lata include parakeratosis with neutrophilic crust, eroded irregular epidermal hyperplasia, hypogranulosis, and superficial pallor [5]. In concordance with these findings, the patient's histopathological examinations from flat papule lesions suspected of being condyloma lata lesions on the face, abdomen, and inguinal revealed eroded epidermis with neutrophil infiltration, spongiotic reactions, acanthosis, parakeratosis, hypogranulosis, superficial pallor, and endarteritis with the infiltration of plasmocytes and polymorphonuclear cells, which support that the patient's lesion is condyloma lata.

The differential diagnosis of the lesions was deep mycoses infection. The histopathological findings did not support the diagnosis, and periodic acid–Schiff staining was performed to rule out the deep mycoses infection with a negative result.

Syphilis is a treatable disease, and secondary syphilis can be treated with a single dose injection of 2,4 million units of BPG intramuscularly. In secondary syphilis, treatment is successful if there is a 4-fold decrease in the VDRL titer at 6–12 months after the therapy in patients without HIV infections. In patients with HIV infection, the serological response is slower; therefore, treatment is successful if there is a 4-fold decrease in the VDRL titer at 24 months after the therapy [24]. Shrivasta et al. [10] reported a case of secondary syphilis with condyloma lata on the unusual sites that were treated with a single dose of 2.4 million units of BPG intramuscular injection, and the lesions cleared within 20 days posttherapy. The patient in this case report was also treated with a single dose of 2.4 million units of BPG intramuscular injection, and the lesions became hyperpigmented macules at 3 months posttherapy, and the VDRL titer decreased 8-fold, from 1:256 to 1:3; therefore, the treatment of secondary syphilis in this patient was considered successful. The patient also received treatment for the HIV infection with ARV FDC consisting of tenofovir, lamivudine, and efavirenz once a day. Unfortunately, the CD4+ T count and HIV viral loads data were not available for this patient, as these tests had not been performed due to the limitations of the facility in the COVID-19 era. Further assessment of these parameters could provide additional insights into the patient's immune status and its potential impact on the observed dermatological findings. This case report showed that condyloma lata lesions can appear as extensive and outside the flexural and anogenital areas. Physicians should be aware of and consider the diagnosis of secondary syphilis in HIV patients with atypical lesions; therefore, histopathological examination should be performed in syphilis patients with atypical lesions.

## 4. Conclusion

Syphilis is easy to treat, but its diagnosis can be challenging due to atypical clinical manifestations that mimic other dermatological conditions. Condyloma lata, a characteristic lesion of secondary syphilis, may present as an extensive lesion in unusual, extragenital sites. Physicians should maintain a high index of suspicion for secondary syphilis in HIV-positive patients with atypical flat papules or plaque-like lesions suggestive of condyloma lata. Therefore, histopathological examination is recommended in such cases to facilitate early diagnosis and prompt treatment.
